# Takotsubo-like syndrome triggered by fludrocortisone overdose for Addison’s disease: a case report

**DOI:** 10.1186/s13256-016-1074-5

**Published:** 2016-10-12

**Authors:** Radu Campean, Matthias Hasun, Claudia Stöllberger, Johannes Bucher, Josef Finsterer, Christoph Schnack, Franz Weidinger

**Affiliations:** 1 Krankenanstalt Rudolfstiftung, Juchgasse 25, A-1030 Wien, Austria; 2 Hartmannspital Krankenhaus, Nikolsdorfer Gasse 26-36, A-1050 Wien, Austria

**Keywords:** Takotsubo cardiomyopathy, Addison’s disease, Hashimoto thyroiditis, Case report

## Abstract

**Background:**

Reversible left ventricular dysfunction, also termed Takotsubo cardiomyopathy, is rarely reported in Addison’s disease after initiation of hormone replacement therapy. The pathogenesis of this cardiomyopathy is unknown.

**Case presentation:**

A 41-year-old white woman with a history of autoimmune Hashimoto thyroiditis diagnosed 3 years earlier and acute adrenal insufficiency diagnosed 3 weeks earlier presented with new onset of heart failure New York Heart Association class IV, which had started shortly after initiation of hormone replacement therapy with hydrocortisone 20 mg/day and fludrocortisone 0.3 mg/day. Nine days before admission she had collapsed because of dizziness and had a cerebral concussion and open fracture of her nasal bone, however, no further investigations were carried out at that time.

A physical examination revealed leg edema, tachycardia, tachypnea, bilateral basal crepitations, and blood pressure 110/70 mmHg. An electrocardiogram showed sinus tachycardia, low voltage, negative T-waves in V_5_ and V_6_ and a corrected QT interval of 590 ms. Echocardiography revealed a reduced left ventricular systolic function with an ejection fraction of 30 %, and septal, apical, and anterior wall akinesia. Cardiac magnetic resonance imaging showed relative enhancement of gadolinium, indicating hyperemia and capillary leakage, and no myocardial scars. Because of the improvement in her cardiac function, lack of cardiovascular risk factors, and lack of signs for ischemia on magnetic resonance imaging, no coronary angiography was carried out. The results of sellar and renal magnetic resonance imaging were normal. Her troponin T was slightly elevated. Bisoprolol and ramipril were started. Her fludrocortisone dose was reduced to 0.05 mg/day. Her electrocardiogram and systolic function, documented by echocardiography and magnetic resonance imaging, normalized within 6 months.

**Conclusions:**

Although we could not exclude coronary artery disease by coronary angiography, her clinical course and instrumental findings suggest Takotsubo cardiomyopathy of the apical type. Fludrocortisone overdosage and increased myocardial vulnerability due to cortisol deficiency might be pathogenetic factors, whereas myocarditis is unlikely. When hormone replacement in patients with Addison’s disease is initiated, cardiac function should be monitored by electrocardiogram and echocardiography.

## Background

Heart failure in Addison’s disease has been described mainly in older patients with ischemic, valvular, or hypertensive heart disease [1]. Reversible left ventricular dysfunction after initiation of hormone therapy has been reported in only nine patients with Addison’s disease, as listed in Table 1 [2–9]. We present a patient with suspected polyglandular autoimmune type II syndrome (PGA II), in whom transient left ventricular dysfunction with heart failure was documented by echocardiography, electrocardiogram (ECG), serum brain natriuretic peptide (BNP) levels, and cardiac magnetic resonance imaging (cMRI).

**Table 1:** Heart failure occurring after initiation of hormone replacement therapy for Addison’s disease

Reference	Age/Sex	Comorbidity	Therapy	Onset of heart failure after	Left ventricular function	Left ventricular function normal after	Acute electrocardiogram	Follow-up electrocardiogram	Heart failure therapy	Outcome
2	47/f	None	HC, FC	20 days	Normal, pericardial effusion	4 months	Normal	NR	Furosemide	R
3	11/m	None	HC	1 day	Severely reduced	6 months	VT	NR	Catecholamines, furosemide	R
4	68/m	Mitral valve prosthesis, hepatopathy	Cortone (cortisone)	4 years	NR	NR	NR	NR	Furosemide, nitrates	R
5	71/f	None	HC	Shortly	Apical ballooning	9 months	ST↑	NR	ß-Blocker, ACE-inhibitor	R
6	69/f	Pyelonephritis	HC	3 days	Severely reduced	3 weeks	T-Inv, QT→, VT	Regression of T-Inv	Cardioversion	NR
7	6/m	None	HC, FC	20 days	Severely reduced	7 days	NR	NR	Furosemide, spironolactone	R
8	42/f	None	HC	2 days	Severely reduced	7 days	ST↑	Normal after 1 week	Catecholamines, mech vent	R
9	53/f	Thyroiditis	HC	Several days	Apical ballooning	2 weeks	ST↑, T-Inv, QT→	NR	NR	R
Present case	41/f	Thyroiditis	HC, FC	3 weeks	Severely reduced	3 months	T-Inv, QT→	ST abnormality	ß-Blocker, ACE-inhibitor, furosemide	R

*ACE* angiotensin-converting enzyme, *f* female*, FC* fludrocortisone, *HC* hydrocortisone, *m* male, *mech vent* mechanical ventilation, *NR* not reported, *QT→* QT prolongation, *R* recovered, *ST↑* ST segment elevation, *T-Inv* T-wave inversion, *VT* ventricular tachycardia

## Case presentation

A 41-year-old white woman presented in December 2014 to our cardiology department with new onset of heart failure New York Heart Association (NYHA) class IV. She reported that she had gained 8 kg weight in the last 4 weeks.

She had a history of a surgically removed uncomplicated ovarian cyst 17 years ago. In 2011, autoimmune Hashimoto thyroiditis was diagnosed and substitution therapy with levothyroxine was started. In November 2014, she was hospitalized in another hospital because of generalized weakness, weight loss of 19 kg, and abdominal pain. Adrenal insufficiency was suspected and confirmed by a positive Synacthen test (Table 2). In view of her autoimmune thyroid disease, the diagnosis of PGA II was suspected. Therapy with levothyroxine 50 μg/day was continued and steroid replacement therapy with Hydrocortone (hydrocortisone) 20 mg/day and fludrocortisone 0.3 mg/day was initiated on 21 November 2014. She was discharged 2 weeks later in a stable clinical condition. Nine days before her actual admission she collapsed because of dizziness and had a cerebral concussion and open fracture of her nasal bone. The cause for the collapse was not further investigated, and neither blood tests, nor cardiac, nor neurological investigations were carried out. As an out-patient she received paracetamol against pain, local therapy with vitamins A and E, and antibiotic therapy with amoxicillin and clavulanate potassium for 7 days. Her family history disclosed that one of her three children, who is now 7-years old, was diagnosed with trisomy 21 and hypothyroidism.

**Table 2:** Results of blood and urine tests

Day	Normal range	18.11. 2014	21.11. 2014	09.12. 2014	17.12. 2014	18.12. 2014	22.12. 2014	19.03. 2015	07.04. 2015
Leukocytes (G/l)	4.0–9.0	7.2	8.3	18.08	11.8	8.7	NM	11.5	11.5
Hemoglobin (g/dl)	12.0–16.0	11.7	9.7	9.0	9.1	9.1	NM	12.4	12.2
Potassium (mmol/L)	3.3–5.1	4.3	3.8	2.99	3.5	4.7	3.8	3.4	3.9
Sodium (mmol/L)	135–150	134	139	144	136	140	141	138	140
Creatinine (mg/dl)	0.50–1.00	0.7	0.8	0.48	0.6	0.55	0.60	0.64	0.66
C-reactive protein (mg/l)	0.0–5.0	0.5	0.2	0.13	0.2	1.5	NM	NM	0.5
NT pro BNP (ng/l)	<116	NM	NM	NM	18.349	NM	14.100	129	NM
Iron (mg/dl)	33–193	57	NM	27	NM	NM	18	86	222
Ferritin (μg/l)	15–150	134	NM	NM	NM	NM	18	39	38
CK (U/l)	26–145	NM	NM	NM	72	41	NM	NM	69
Troponin T (ng/ml)	0.000–0.009	NM	NM	NM	0.014	NM	0.007	NM	NM
TSH (μU/ml)	0.200–3.700	1.14	1.84	NM	9.06	5.94	NM	NM	5.31
Serum cortisol (μg/dl)	1–18	0.2	NM	NM	23.7	NM	NM	NM	25.08
ACTH (pg/ml)	<46	1310	NM	NM	NM	NM	NM	NM	NM
PTH (pg/ml)	14.9–56.9	NM	NM	NM	NM	NM	62.3	NM	43.0
Cortisol excretion (nmol/24 hours)	57.7–806.8	NM	NM	NM	NM	NM	1492.66	NM	NM
Serum renin (pg/ml)	2.90–27.60	NM	0.73	NM	0.36	NM	NM	NM	NM

*ACTH* adrenocorticotropic hormone, *CK* creatine kinase, *NT pro BNP* N-terminal prohormone of brain natriuretic peptide, *NM* not measured, *PTH* parathyroid hormone, *TSH* thyroid-stimulating hormone

**Fig. 1:**
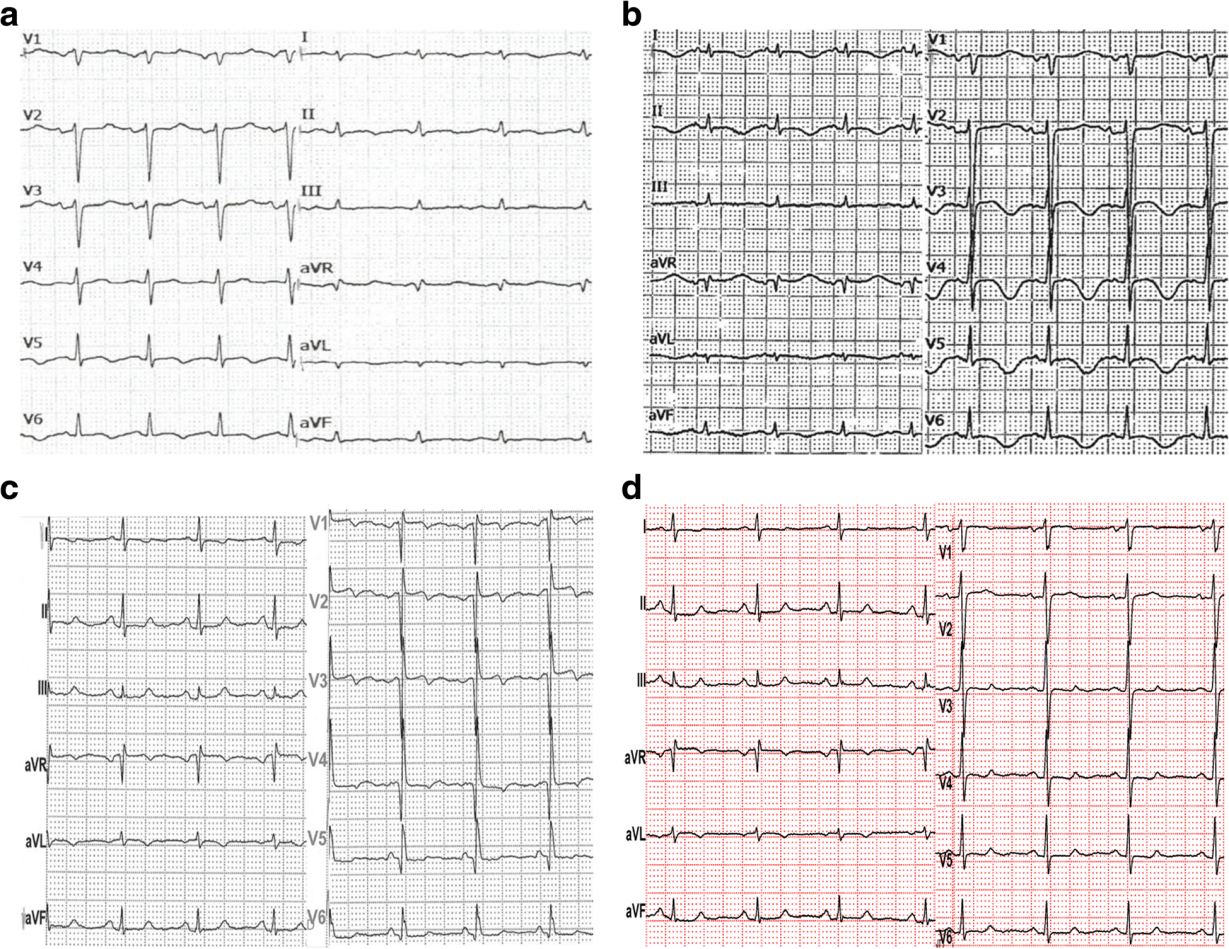
Time course of electrocardiogram. **a** 16 December 2014: on the day of admission, only slight T-wave abnormalities are present. **b** 18 December 2014: 2 days later, negative T-waves are present in leads I, II aVL, and aVF V_3–6_, and the corrected QT interval is prolonged (590 ms). **c** 19 March 2015: 3 months later, corrected QT interval has normalized and only slight ST abnormalities are seen in leads I, aVL, and V_3–6_. **d** 9 July 2015: after 6 months, there is only persistence of a negative T-wave in lead aVL, and the ST abnormalities have regressed

**Fig. 2:**
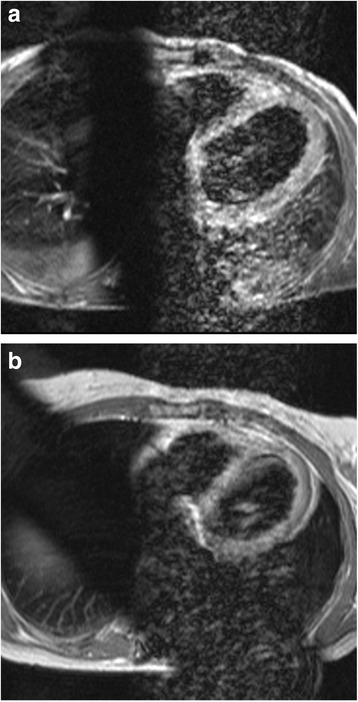
Time course of cardiac magnetic resonance imaging. **a** 23 December 2014: cardiac magnetic resonance four-chamber view showing relative enhancement of the left ventricle post-contrast media administration with an elevated T1-ratio of 9.05 (normal range <4) indicating hyperemia and capillary leakage. **b** 3 June 2015: cardiac magnetic resonance four-chamber view showing a normal T1-ratio of 2.25 (normal range <4)

On admission, a physical examination showed orthopnea, blood pressure 110/70 mmHg, tachycardia of 102 beats per minute (bpm), tachypnea of 30 breaths/minute, bilateral basal crepitations, and leg edema. Her oxygen saturation was 90 % at rest, without oxygen. An ECG revealed a sinus tachycardia, low voltage, negative T-waves in V_5_ and V_6_ and a c corrected QT interval (QTc) of 590 ms (Fig. 1). Her serum troponin T levels were only slightly elevated. The results of blood and urine tests are listed in Table 2. Echocardiography showed septal, apical, and anterior wall akinesia and a severely reduced left ventricular systolic function with a left ventricular ejection fraction (EF) of 30 %. CMRI confirmed her highly reduced systolic function with a left ventricular EF of 28 % and showed a relative enhancement, indicating hyperemia and capillary leakage (Fig. 2). Neither ischemic areas, nor myocardial scars, nor fibrotic changes were detected. Sellar and renal magnetic resonance imaging (MRI) investigations were normal.

Therapy with bisoprolol and ramipril, and furosemide administered intravenously was initiated. Her fludrocortisone dose was reduced to 0.05 mg/day. Under that therapy, her clinical condition improved, her BNP levels regressed (Table 2), echocardiography showed an improvement of systolic function, and ECG abnormalities regressed (Fig. 1). Because of the improvement in her cardiac function, lack of cardiovascular risk factors, and lack of signs for ischemia on MRI, no coronary angiography was carried out. She was discharged as heart failure NYHA class I after 2 weeks with a pharmacotherapy comprising Hydrocortone (hydrocortisone) 20 mg/day, fludrocortisone 0.05 mg/day, levothyroxine 50 μg/day, pantoprazole 40 mg/day, bisoprolol 5 mg/day, and ramipril 10 mg/day. Follow-up investigations 6 months later showed normalization of her ECG and systolic function, documented by echocardiography (Fig. 1) and cMRI (Fig. 2).

## Discussion

In our case, heart failure occurred 3 years after the diagnosis of thyroiditis, 3 weeks after the diagnosis of Addison’s disease and initiation of the hormonal therapy, and 9 days after her collapse, when her condition was slowly normalizing. According to previous reports and her history, there were no indications for preexisting cardiovascular disorders.

The pathogenesis of reversible systolic dysfunction in Addison’s disease, resembling Takotsubo cardiomyopathy (TTC), is unknown. There are several speculations about the pathophysiologic mechanism of heart failure after initiation of hormone therapy in Addison’s disease. One speculation assumes that once mineralocorticoid replacement therapy is commenced, renal adaptation to chronic water and sodium loss leads to overshooting of water and sodium retention with ensuing heart failure [2]. Development of heart failure with fludrocortisone therapy was reported for the first time in a series of adults with Addison’s disease [1]. Patients with heart failure from that series, however, had preexisting ischemic, valvular, or hypertensive heart disease and were older patients. In these patients, heart failure was managed with a dose reduction or cessation of fludrocortisone [1].

Also without preexisting cardiovascular disease, mineralocorticoid overdose has been implicated in ventricular dysfunction in Addison’s disease (Table 1) [2, 4, 7]. It remains unclear why our patient received an unusually high dose of fludrocortisone 300 μg/day, three times more than recommended [10]. She had gained 8 kg of weight after initiation of therapy, and heart failure regressed with reduction of fludrocortisone.

However, her clinical presentation was not compatible with pure volume overload. She showed ECG changes similar to those of myocardial ischemia, albeit the absence of cardiovascular risk factors, her clinical course, and her normal levels of creatine kinase with only slight elevations of troponin and no indications for myocardial ischemia on repeated cardiac MRIs virtually ruled out this possibility. The findings and her clinical course resemble apical TTC, characterized by symptoms and ECG signs of acute myocardial infarction, transient left ventricular dysfunction but without angiographic evidence of coronary stenosis [11]. Since coronary angiography was not carried out, we cannot establish definitively the diagnosis of TTC. Different acute psychic and physical triggers of TTC, including endocrine disorders like Addison’s disease, have been described [12]. Traumatic head injury with concussion and nasal bone fracture, which had occurred 9 days before admission, could have been a major stressor event, additionally triggering TTC.

Regarding the pathomechanism of TTC in Addison’s disease, there are only animal data available. Studies in adrenalectomized animals suggest that cardiac function may be markedly impaired when stress is superimposed, and increased catecholamine levels are toxic to a myocardium that is unprotected by glucocorticoids [13]. Glucocorticoids have been shown to be important for the maintenance of membrane calcium transport function in the cardiac sarcoplasmic reticulum from the rat, and may therefore affect myocardial contractility [14]. Microsomal phosphorylase activity in rat heart muscle is depleted by adrenalectomy. The decreased phosphorylase activity may impair glycogenolysis and thus induce derangement of excitation–contraction coupling of the heart [15]. It was speculated that cortisone deficiency might lead to myocardial injury and that probably hydrocortisone can exert a toxic effect on a previously cortisone-starved myocardium [8]. Furthermore, it can be assumed that rather than a direct toxic role of steroids, a greater sensitivity to the adrenergic system induced by the administration of steroids may play a role in the pathogenesis of reversible left ventricular dysfunction.

Myocarditis could be another explanation for the transient left ventricular dysfunction. However, neither history, nor blood tests, nor MRI were indicative of myocarditis [16]. A further pathomechanism might be a transient affection of the myocardium by an underlying autoimmune disorder. In our patient, her systolic function recovered, whereas in a previously described patient with PGA II, the autoimmune cardiomyopathy required heart transplantation [17].

The reason for her collapse that occurred 9 days before her actual admission remains unclear. Since her serum potassium level was low at the actual admission (Table 2), it can be assumed that hypokalemia due to fludrocortisone overdose might have played a role, either by generalized muscle weakness or by inducing arrhythmia [18]. A further cause for the collapse could be arrhythmia due to QT prolongation; however, no ECG had been recorded at the time of her collapse.

Limitations of the presented case are that no coronary angiography was carried out to establish the diagnosis of TTC and no myocardial biopsy was carried out to definitively exclude myocarditis, thus the case does not fulfill all the diagnostic requirements for TTC according to the Mayo Clinic diagnostic criteria [11]. Furthermore, the cause of our patient’s collapse which occurred 9 days before her actual admission remains unclear and no ECG recordings are available from the time when her symptoms started. Thus it cannot be assessed whether she also showed ST elevations which are reported to occur less frequently in patients with TTC than in acute coronary syndromes [19].

## Conclusions

Patients with Addison’s disease should be monitored for cardiac abnormalities during therapy for acute adrenal insufficiency. If they develop symptoms suggestive for arrhythmia, their serum electrolytes should be measured and an ECG should be recorded. If they develop heart failure or left ventricular dysfunction, hormonal therapy should be considered to be a possible iatrogenic trigger.

## Abbreviations

BNP: Brain natriuretic peptide
cMRI: Cardiac magnetic resonance imaging
ECG: Electrocardiogram
EF: Ejection fraction
MRI: Magnetic resonance imaging
NYHA: New York Heart Association
PGA II: Polyglandular autoimmune type II syndrome
QTc: Corrected QT interval
TTC: Takotsubo cardiomyopathy
